# Breast Health Training Program for Traditional Healers: A Pathway to Early Breast Cancer Detection in Tanzania

**DOI:** 10.1002/cnr2.70377

**Published:** 2026-01-14

**Authors:** Autumn Beavers, Elizabeth F. Msoka, Irene Masue, Theresia Mwakyembe Mwansasu, Ayesiga Herman, Blandina T. Mmbaga, Lily Gutnik

**Affiliations:** ^1^ The University of Alabama at Birmingham Birmingham Alabama USA; ^2^ Kilimanjaro Clinical Research Institute Moshi Tanzania; ^3^ Kilimanjaro Christian Medical Centre Moshi Tanzania

**Keywords:** breast cancer, clinical breast examination, referral pathway, traditional healers, training program

## Abstract

**Background:**

In sub‐Saharan Africa, traditional healers often serve as primary healthcare providers and are the first point of contact for patients. Given this, they are uniquely positioned to aid in early breast cancer detection. To evaluate this, we implemented a breast cancer training program to equip traditional healers in Tanzania with foundational knowledge and CBE skills, aiming to improve early detection and timely treatment.

**Aims:**

To implement a breast cancer training program to equip traditional healers in Tanzania with foundational knowledge and CBE skills, aiming to improve early detection and timely treatment.

**Methods:**

We conducted a breast cancer training program in Tanzania among rural registered traditional healers. Knowledge acquisition was assessed through pretest and posttest surveys.

**Results:**

Three male and three female rural traditional healers (average age: 53) participated in the training, with 67% having no prior formal breast cancer training, though 83% reported some breast cancer awareness. Pre‐ and post‐training assessments showed an increase in breast cancer knowledge, with median scores rising from 41% to 74% (*p* = 0.01). All participants found the training valuable and felt empowered to serve as community health advocates, with widespread consensus that they would prioritize referring patients with suspicious breast findings to the hospital rather than attempting to treat.

**Conclusion:**

Traditional healers, as trusted community figures and primary healthcare providers, offer a promising solution to bridging gaps in early breast cancer detection in Tanzania. Enhancing their breast cancer knowledge and equipping them with the skills to identify suspicious breast findings provides a scalable strategy for improving early detection in resource‐limited settings.

## 1 Introduction

The global burden of breast cancer is increasing worldwide, with an estimated 2.3 million new cases diagnosed and 670,000 deaths reported in 2022 [1]. Low‐ and middle‐income countries (LMICs) face disproportionately higher mortality rates despite lower incidence, primarily due to late‐stage presentation [2], which is often driven by prolonged diagnostic delays [3]. Espina et al. found that women in North and sub‐Saharan Africa experienced delays of 4–15 months between recognizing symptoms and receiving a diagnosis [3]. In Tanzania, over 80% of breast cancer patients are diagnosed at stage III or IV [4]. Barriers to early detection include limited accessibility to screening programs, low breast cancer awareness among communities and healthcare providers, widespread misconceptions, and initial reliance on traditional medicine [5, 6, 7].

Traditional healers (THs) play a vital role in the healthcare systems of sub‐Saharan Africa, with up to 80% of the population utilizing their services [8]. This reliance is driven by cultural norms, perceptions of safety and effectiveness, and the accessibility and affordability of traditional medicine [9, 10, 11, 12]. Research has shown THs are involved across the cancer care continuum, primarily in the treatment phase [7]. However, the methods they use for diagnosis and the reasoning behind their treatment decisions remain unclear. In Tanzania, many women with breast symptoms initially seek care from THs, often spending over 3 months on alternative treatments [4]. This delay is influenced by strong patient–healer trust, cultural beliefs, and perceptions that symptoms may be due to supernatural causes, such as bewitchment [4]. Women will often continue with traditional remedies until their symptoms worsen, at which point they seek biomedical care [4]. This represents a critical missed opportunity for early detection, as it increases the risk of advanced disease presentation.

Given that THs are often the first point of contact for patients, improving their breast health knowledge and skills may reduce misdiagnoses and facilitate timely referrals. Several programs across Africa, including Tanzania, have shown that with proper training, THs can implement culturally appropriate, evidence‐based practices [13, 14, 15]. In the Democratic Republic of Congo, Congo‐Brazzaville, THs were trained to identify and refer suspected cases of Human African trypanosomiasis, resulting in 278 referrals, 20 positive screenings, and 12 confirmed diagnoses [16]. In Tanzania, Africa Centres for Disease Control (CDC) trained 96 THs to recognize Marburg Virus symptoms, combat misinformation, and coordinate with health authorities to improve infection prevention and control [17]. Additionally, the Eye Care Foundation piloted a project in Morogoro to train THs to identify eye disorders early and refer patients to appropriate services [18]. Collectively, these examples highlight that when equipped with targeted training, THs can be pivotal partners in improving early detection and linkage to biomedical care. This suggests that similar models could be adapted for breast cancer, offering a practical, community‐based solution to addressing gaps in breast cancer detection.

Preliminary findings from an ongoing study at the study site, Kilimanjaro Christian Medical Centre (KCMC), demonstrated THs' desire to improve their breast health knowledge and skills and their willingness to refer patients to the hospital [19]. Building on this, we conducted a study to train THs in foundational breast health and cancer knowledge, including how to perform a CBE, and establish a referral system for patients with suspicious breast concerns in Moshi, Tanzania. To our knowledge, this is the first study to implement and evaluate a structured breast health training program for THs. This present manuscript focuses on the training component, describing its design and delivery, and assessing immediate post‐training knowledge and skill outcomes.

## 2 Methods

### 2.1 Study Setting

This study was conducted at Kilimanjaro Christian Medical College (KCMC), located in Moshi, the capital of the Kilimanjaro region in Tanzania. KCMC is one of Tanzania's four zonal consultant hospitals and a key center for medical services, research, education, and healthcare delivery in East Africa [20]. With approximately 630 beds, it serves as a referral hospital for over 15 million people across northern, eastern, and central Tanzania, including Kilimanjaro, Arusha, Tanga, Singida, and Manyara [20]. Kilimanjaro Clinical Research Institute (KCRI) and the KCMC Cancer Care Center (CCC), both based at KCMC, are leading institutions in cancer care in Tanzania and the Moshi region. KCRI is an academic center dedicated to evidence‐based health interventions and has significantly expanded over four decades, becoming one of the leading research facilities in Tanzania [21]. CCC is one of three comprehensive cancer centers in the country. Managing over 900 new cases annually, it serves both adult and pediatric patients, with 65% requiring radiation therapy [21]. A Cancer Radiation Center, set to open in mid‐2025, will significantly expand services, addressing a major gap in Tanzania's cancer care.

### 2.2 Recruitment

Due to the study team's established relationship with the Traditional and Alternative Medicine Regional Coordinator through previous studies and her familiarity with THs in the region, she was chosen to assist in recruiting additional THs. Six healers were selected by the Regional Coordinator, who is appointed by the Council for Traditional and Alternative Medicine Practitioners (CTAMP), and is responsible for enforcing regulations, supporting practitioners, and monitoring compliance as outlined in The Traditional and Alternative Medicine Act [22]. Selection was based on the healers' willingness to participate, involvement in the local chapter, and adherence to organizational rules and regulations. All participants were required to meet the following criteria: be registered under the Traditional and Alternative Health Practice Council in Tanzania, be at least 18 years old, complete a 5‐day breast health and cancer training course, be free of visual impairments, and have the ability to read and write in Swahili. Training took place in a conference room of KCRI for 5–7 h daily.

### 2.3 Training Components

#### 2.3.1 Breast Cancer Attitudes and Beliefs

A half‐day roundtable discussion was held to understand better THs' perceptions of breast cancer diagnosis and treatment in Tanzania. The discussion explored how THs diagnose and treat breast cancer, the costs patients incur for treatment, and their perceived role in cancer care.

#### 2.3.2 Breast Cancer Knowledge

A breast health worker training program previously described in Malawi for laywomen was adapted for this cohort [23]. The training was conducted over a 5‐day period and covered topics such as breast anatomy and physiology, common benign breast conditions, and breast cancer screening, diagnosis, and treatment. PowerPoint presentations were used as the primary teaching format in combination with hands‐on experience for CBE training using a donated simulation model. Each TH received a three‐ring binder containing all training materials, including an in‐depth manual on breast cancer and a printed version of the PowerPoint slides in Swahili. Multiple‐choice questions were incorporated into the PowerPoint presentation and posed immediately after each lecture. This approach assessed participants' understanding of the material in real time and encouraged further discussions or questions related to the topic to assure the concepts were well understood. Each morning began with a review session to reinforce the previous day's material, ensuring a solid foundation of knowledge, as each day's content was designed to build on the last. The pre‐intervention survey assessed baseline knowledge. Due to challenges faced by participants in understanding the questions and testing format of the pretest, the study team read each question aloud, and participants independently marked their responses. Further explanation was provided as needed to ensure clarity and comprehension. After the training, THs completed the 37‐question post‐training survey to evaluate knowledge acquisition and the training program's effectiveness. The survey included 32 Likert‐scale questions addressing breast cancer signs, symptoms, risk factors, epidemiology, and treatment. All training sessions and discussions were conducted in real time with translation into Swahili to ensure comprehension and active engagement.

#### 2.3.3 Clinical Breast Exam

THs were trained to perform CBEs via didactics and simulators. Participants independently practiced the exam using the Nasco Life/Form Advanced Breast Exam Simulator, with each participant receiving individual guidance from a fellowship‐trained breast surgeon. They were trained to visually inspect breasts for asymmetry, visible lumps, skin changes, nipple retraction, discharge, or axillary swellings, first with the woman in an upright position (hands on hips) and then in the supine position. For palpation, they were taught to use the pads of their middle three fingers, performing overlapping dime‐sized circular movements and working from the outermost part of the breast toward the nipple. Palpation of axillary and supraclavicular lymph nodes was taught with the woman in an upright position. Additionally, they were instructed on how to properly document their exam findings to ensure accurate and consistent records.

#### 2.3.4 Hospital Tour

The healers participated in a personalized hospital tour designed to familiarize them with the multidisciplinary care involved in breast cancer management and to provide knowledge they could relay to their patients. They were guided through the standard workflow for a patient presenting with a breast concern, covering initial evaluation, diagnostic testing, and treatment pathways. During the tour, they met with key healthcare providers, including a hematologist/oncologist, pathologist, and breast surgeon. They visited essential departments such as the breast surgery clinic, ultrasound, mammography, computed tomography (CT), pathology, hematology/oncology (chemotherapy), and radiation oncology. The small group setting allowed healers to engage in detailed discussions, ask specific questions about breast cancer diagnosis and treatment, and address common myths and misconceptions.

### 2.4 Data Collection

#### 2.4.1 Training Evaluation

A post‐training survey was conducted to evaluate their experience, as well as assess the likelihood of healers referring patients with suspicious breast concerns. Additionally, three open‐ended questions explored how the training might influence their practice, which topics they found most valuable, and suggestions for content they felt should be included in future sessions.

#### 2.4.2 Data Analysis

Data were analyzed using Microsoft Excel. Variables were compared using independent two‐tailed *t*‐tests assuming equal variances, with statistical significance set at *p* < 0.05.

#### 2.4.3 Ethics Statement

Ethical approval for this study was obtained in Tanzania through a two‐tier process. Initial review and clearance were granted by the KCMC Research Ethics Committee, followed by approval from the National Health Research Ethics Committee (NatHREC) under the National Institute for Medical Research (NIMR). All protocols were submitted via the national online Research Ethics Information Management System, and, because the study involved international collaboration, a research permit was also secured from the Tanzania Commission for Science and Technology (COSTECH). Additional ethical approval was obtained from the University of Alabama at Birmingham, the corresponding author's home institution, through its standard process involving submission to the Institutional Review Board (IRB) for review, risk assessment, and issuance of formal approval prior to study initiation.

## 3 Results

### 3.1 TH Characteristics

All six THs were recruited from the Kilimanjaro Region, with a median age of 53 (range 43–78). Among the participants, 67% (*n* = 4) had never attended formal training from a medical professional, though 83% (*n* = 5) reported prior breast cancer awareness. Of those who reported prior breast cancer awareness, 67% indicated that their primary sources of information were radio and television. The cohort was evenly split by gender (50% women, 50% men), and all participants identified as Muslim and resided in rural areas of the Kilimanjaro Region. All participants' highest level of education was primary school, and 50% were married. The average monthly income was approximately 246,000 Tanzanian Shillings (98 USD) (Table 1). Notably, 100% of their practices were inherited from family members. Half of the participants treated 30–50 patients weekly, with their primary focus being on noncommunicable diseases, reproductive health, and infertility. All participants reported treating various cancer types using oral and topical approaches, with the typical treatment course lasting about 3 months.

**TABLE 1:** Traditional healers characteristics.

TH	Age	Sex	Marital status	Level of education	Years in practice	Practice location	Prior breast cancer knowledge	Sources of breast cancer information
1	56	F	Married	Primary school	11–20	Rural	Yes	Radio, internet
2	63	F	Widowed	Primary school	> 20	Rural	Yes	Family, radio, TV, health center, seminar
3	43	F	Single	Primary school	0–5	Rural	No	Not applicable^a^
4	78	M	Married	Primary school	> 20	Rural	Yes	Radio, TV, health center
5	50	M	Married	Primary school	> 20	Rural	Yes	TV, seminar
6	49	M	Divorced	Primary school	> 20	Rural	Yes	Family, radio, TV

^a^ Participant reported no prior breast cancer awareness.

### 3.2 Pre‐ and Post‐Course Survey

Breast cancer knowledge scores improved between pre‐ and post‐course surveys. Median overall correct score increased from 41% (pretest) to 74% (post‐test) (*p* = 0.01), with one participant's score rising from 0% to 78% (Table 2). The greatest knowledge gains were observed in breast cancer risk factors and treatment. Knowledge of treatment modalities, including surgery, chemotherapy, and hormonal therapy, increased to ≥ 83%. Post‐training, all participants correctly marked that breast cancer is treatable when detected early and a leading cause of death in Tanzania. However, misconceptions, such as the belief that bras or storing money or phones in bras cause breast cancer, remained unchanged or only slightly improved. Notably, the belief in traditional medicine as a treatment option for breast cancer slightly decreased from 83% to 67% among healers.

**TABLE 2:** Pre and post survey test results.

Breast cancer content (# of questions)	TH 1		TH 2		TH 3		TH 4		TH 5		TH 6		All	
	% correct													
	Pre	Post	Pre	Post	Pre	Post	Pre	Post	Pre	Post	Pre	Post	Pre	Post
Risk factors (12)	25	67	50	83	0	100	58	67	17	58	25	75	29	75
Signs and symptoms (12)	58	58	58	92	0	50	67	67	67	58	67	75	49	68
Epidemiology (3)	67	100	100	100	0	67	67	67	100	100	100	100	55	88
Treatment (5)	40	60	100	100	0	100	40	80	20	80	0	80	30	80

Abbreviation: TH = traditional healer.

### 3.3 Lecture Questions

Multiple‐choice questions during the didactic sessions at the end of each topic presentation revealed variability in participants' understanding of breast health topics (Table 3). Breast knowledge was strong for topics such as the function of breast lobes (83% correct) and common causes of breast infection (100% correct) but limited for foundational concepts like the structures of the nipple‐areola complex and natural changes in older women's breasts (0% correct). Regarding breast cancer, knowledge was high for understanding breast cancer behavior (83% correct) but limited for topics such as how breast cancer spreads and the factors influencing its spread (17%–33% correct). In the signs and symptoms of breast cancer category, participants were moderately successful in identifying important clinical signs (67% correct); however, they demonstrated a limited understanding of breast cancer staging and clinical presentation (17% correct). Knowledge of breast cancer treatment was generally low, with correct responses ranging from 17% to 33% across topics such as treatment aims, local management, and the timing of hormone therapy.

**TABLE 3:** Traditional healer lecture questions.

	All % correct
Breast structure and function	
Structures of the nipple‐areola complex	0
Function of breast lobes	83
Size of breast in non‐breastfeeding woman	83
Neck lymphatic drainage	50
Menstrual cycle	67
Breast changes during pregnancy	17
Natural changes in older women's breast	0
Benign breast	
Signs of fibroadenoma	33
Clinical findings of breast cysts	0
Most common patient population for fibroadenomas	83
Physical exam findings of fibroadenoma	33
Causes of breast cysts	83
Age of presentation for breast cysts	33
Common cause of breast infections	100
Breast cancer	
How breast cancer spreads	17
Factors that determine whether breast cancer is likely to spread	33
How breast cancer behaves	83
Signs and symptoms of breast cancer	
Definition of breast cancer staging	17
Clinical presentation of breast cancer	17
Important clinical signs of breast cancer	67
Breast cancer treatment	
Aim of breast cancer treatment	17
Types of local breast cancer management	33
When to give hormone therapy based on hormone receptor status	17

### 3.4 TH Inquiries

During each didactic session, the THs posed a range of practical questions about breast health and breast cancer, highlighting key areas of interest and gaps in knowledge. This led to vibrant discussions among the trainers and healers. Their questions were organized into thematic categories, including benign breast conditions, general breast health, breast cancer knowledge, breast cancer risk factors, pregnancy and breast cancer, cancer myths and misconceptions, cancer treatment, the referral process, clinical breast examination, patient barriers to referral, and cancer trends in Tanzania (Table 4). A significant portion of questions centered on breast cancer, particularly on recognizing its signs and symptoms. For example, participants asked, “If there is no [breast] lump but nipple discharge, is this concerning for breast cancer?” and “What are the different types of breast cancer?” Questions about breast cancer biology and heredity were also common, including inquiries about whether breast cancer can affect both breasts and whether it is hereditary. There was significant interest in the relationship between pregnancy, breastfeeding, and breast cancer. Questions such as, “If a woman is breastfeeding and has cancer, can the child also get breast cancer?” and “What should a woman do if she has a lump during pregnancy?” were asked. Patient financial and logistical barriers to care were also prominent themes. Participants expressed concerns about the affordability of diagnostic tools and treatment, asking, “Who will pay for patients who need to come to the hospital but do not have money?” and “If a mammogram is expensive, how can poor people access it?” Finally, participants showed a keen interest in breast cancer trends in Tanzania. Participants inquired about the government's stance on the rise in breast cancer cases, potential links to dietary changes, and strategies to reduce cancer rates.

**TABLE 4:** Traditional healers' questions themes.

Themes	Questions
Benign	What is the relationship between fatty tissue and the milk producing part?
	Where does lymph node drainage go? Can it be helped with medications
	A patient had swollen cervical lymph nodes and were given medications, and the swelling moved to breast and then went away—what could that be?
General breast health	Is there a proper bra that women should wear?
	Many women do not like to wear bras, can you comment on this?
	Do women with naturally small breast still need a bra?
General breast cancer knowledge	If there is no lump but there is nipple discharge, is this concerning for breast cancer?
	Is discharge from both breasts concerning for breast cancer?
	Can breast cancer present as a neck mass that moves in the breast after some time?
	If there are bilateral breast concerns, that means there is no cancer, correct?
	If there is nipple discharge and pain in axilla but there is no mass, is this still breast cancer?
	Is breast cancer hereditary?
	Is it possible to see breast cancer in both breasts?
	Is breast milk part of cancer?
	What are the different types of breast cancer?
	Are there other antennas [receptors] on the cell other than the ones presented?
	If a woman does not have all or any of the antennas [receptors], does this mean she does not have breast cancer?
	Is there any prevention of breast cancer?
Breast cancer risk factors	Are oral contraceptives a cause of breast cancer?
	Can breast cancer be passed down in families?
	Is a woman's menses a risk factor for BC?
	If a woman who has no problems with her menstrual cycle decides to have a child after the age of 30, can she still develop breast cancer?
Pregnancy and breast cancer	Why do some children refuse the breast completely when breastfeeding?
	What should we do if a woman breastfeeding has breast pain?
	What if a woman has a lump during pregnancy? What should she do?
	If a woman is breastfeeding and has cancer, can the child also get breast cancer?
	If a woman breastfeeds and after breast feeding the child develops diarrhea or vomit that is the color of breast milk, what is the relation to breast cancer?
	If pregnant women have full breast and pain, is this concerning for breast cancer?
	If a woman is pregnant and has cancer, can this be transmitted to the baby?
Cancer myths and misconceptions	Is it possible if someone has cervical cancer that it can become breast cancer?
	If someone uses a razor blade that has blood on it from a person with breast cancer on their breast or skin, can they get breast cancer?
	For growing girls who are about to get their period, if they are keeping money or phones in the breast, is this causing breast cancer?
	Young girls who get abortions or girls who throw their babies away after birth and get hormonal injections to prevent their breast from forming milk, does this cause breast cancer?
	Should men breastfeed from women? This has been advertised on the radio.
Cancer treatment	If a patient has diabetes and breast cancer, how will removing her breast affect this? Will the wound be able to heal after surgery?
Referral process	Once we go back to our offices and find something concerning on exam, instead of giving them medication, should we send them to the hospital first?
	We will refer all our patients who we are concerned about, but is there a way doctors can also refer patients to us?
	Will it be possible to know who the doctor treating our patient is? And can the patient be referred back to us before surgery so that we can treat them?
Clinical breast exam	When you are examining the patient with your fingers, will the patient feel pain?
	If I find a lump, what should I do?
	Can men be checked the same way as women?
	When we say a woman should lift her hands [during a breast exam] and put them on her hips, is it okay for pregnant women to also do this?
	If a woman is in her 3rd trimester pregnant, is it okay for her do this [lift her hands above her head]?
Patient barriers to referral	Who will pay for patients who come to the hospital?
	What should we do for patients who go to the hospital but are afraid to go back? How should we counsel them?
	Who will pay for patients who need to come to the hospital but do not have money?
	If a mammogram is expensive, how can poor people have access to it?

### 3.5 Training Evaluation

Post‐training surveys and group discussions provided valuable insights into the impact of the training on THs. Several key themes emerged, including a newfound acknowledgment of the role THs play in “holding” patients and delaying treatment, with one healer stating, “We were trying to treat something we did not understand and now see that by holding those patients, we contributed to the advanced stage of disease.” Another reflected, “I do not want to hate myself as a traditional healer, but I now really understand that we have to work with the doctors to eradicate this disease.” The hands‐on CBE training and hospital tour were identified as the most impactful components of the program, with one healer remarking, “I did not realize how detailed it [breast cancer care] was and how so many doctors work together to take care of the patient.” Many healers expressed a desire to continue collaborating with the biomedical healthcare system, not only for breast cancer but for other types of cancers as well. Additionally, 100% of participants found the training valuable and felt empowered to serve as community health advocates, with widespread consensus that they would prioritize referring patients to the hospital rather than attempting to treat.

## 4 Discussion

The high burden of late‐stage breast cancer and associated mortality in this region underscores the need for innovative early‐detection strategies. This portion of our study demonstrates that THs, who are deeply embedded within Tanzania's healthcare landscape, can be effectively trained to improve breast health knowledge and collaborate with the biomedical system. With appropriate training, THs can conduct basic clinical assessments, serve as community health ambassadors, and play a critical role in dispelling myths, addressing misconceptions, and reinforcing the importance of breast cancer screening and early diagnosis.

This study builds on prior research demonstrating the effectiveness of involving THs in patient care and service delivery. Historically, healers have been successfully engaged in public health campaigns, including HIV/AIDS, tuberculosis, and Ebola [13, 14, 15, 24]. A cluster‐randomized trial in Uganda evaluating HIV point‐of‐care testing found that 100% of clients who received testing directly from healers underwent HIV testing, compared to 23% in the control group, who were referred to clinics [25]. This finding underscores the potential impact of integrating THs into healthcare interventions to improve patient engagement and access to essential services.

THs were taught basic knowledge of general breast health, cancer risk factors, symptoms, and treatment. Despite low education levels of THs, pre‐ and post‐test results revealed improvements in breast cancer knowledge; however, we suspect their actual knowledge gain may have been even greater than reflected in formal assessments. Many healers posed insightful and thoughtful questions, demonstrating their understanding of key concepts, though this was not always reflected in written assessments. They performed better when tested through oral questioning but faced challenges with lecture and survey questions. It remains unclear whether these difficulties stemmed from their education level or the wording, style, and format of the questions. Additionally, healers actively engaged with one another, answering each other's questions and reinforcing their learning through discussion. Follow‐up discussions with the healers conducted 1 and 2 months post training showed strong retention of key concepts. Many healers not only retained the information but also used it to educate their clients, demonstrating the lasting impact of the training. These results align with prior studies that have shown the feasibility of improving the medical knowledge base of nonmedical community members [13, 14, 15].

THs demonstrated increased awareness of evidence‐based interventions, such as the importance of early detection and the availability of curative treatments for early‐stage breast cancer. However, myths and misconceptions surrounding breast cancer showed minimal improvement, despite frequent discussions, indicating the deeply ingrained nature of such beliefs. Beyond improving knowledge, TH also embraced their roles as community educators and advocates. THs organically established a weekly meeting to discuss challenges, share insights, and reinforce their training, highlighting their proactive commitment to improving community health and fostering a collaborative environment.

The framework developed through this training can be adapted for similar resource‐limited settings to address other health challenges. However, further research is needed to optimize and standardize training curricula for THs, as well as develop continuous education programs to reinforce knowledge and skills and address evolving needs in cancer care. Developing scalable, culturally sensitive programs can help expand such initiatives in settings where healthcare workforce shortages persist. Participants' questions during training highlighted broader systemic issues, including logistical and financial barriers to accessing diagnostic and treatment services. These concerns underscore the importance of developing comprehensive interventions that address not only knowledge and skills but also structural barriers to care. Studies exploring long‐term outcomes of TH training, such as patient referral rates, cancer staging at diagnosis, and treatment outcomes, are essential to evaluate the sustained impact of these programs.

The most significant limitation of this study is the small sample size, which substantially restricts the generalizability of the findings. All participants had demonstrated interest and willingness to collaborate with the biomedical healthcare system, thus demonstrating some degree of selection bias, further limiting applicability to healers who may be less motivated or less engaged in such efforts. This training was conducted with a small group, which allowed for more personalized interactions and enabled us to address individual challenges and specific needs. However, this approach may not be representative of larger‐scale programs. Addressing this limitation will be critical in future research, which should also incorporate measures of post‐training behavioral change and examine how healer–biomedical affiliations influence patient pathways to care. Additionally, the training did not dispel persistent community myths, such as beliefs that wearing a bra or placing a cell phone in the bra increases breast cancer risk, and no formal evaluation of clinical breast examination skills was conducted. Addressing these gaps will be important to fully understand the long‐term impact of similar programs. THs were compensated for their participation, which may have served as an incentive. The training spanned 5 days, each lasting 5–7 h. This structure may have been more feasible for healers with flexible schedules, but it could pose challenges for those who depend on daily income or lack the flexibility to attend extended training sessions. While this study demonstrated immediate post‐training improvements, the impact was assessed only at the conclusion of the 5‐day training. The subsequent 6‐month implementation phase, including referral data and longitudinal outcomes, will be reported in a separate manuscript with preliminary results showing substantially higher referral rates post training. Nevertheless, the limited duration of the training may not be sufficient to achieve enduring practice change. Future programs should explore extended training formats, incorporate refresher sessions to reinforce knowledge and skills, and include long‐term follow‐up to better evaluate the training effects on referral patterns and healer behavior.

## 5 Conclusion

This work highlights the potential for training THs as a cost‐effective and culturally sensitive strategy to improve early breast cancer detection in Tanzania. By addressing knowledge gaps and fostering collaboration between traditional and biomedical healthcare systems, such initiatives can play a pivotal role in reducing delays in breast cancer diagnosis and treatment. Building on this framework and scaling these efforts could enhance healthcare accessibility, reduce diagnostic delays, and improve patient outcomes in LMICs.

## Author Contributions

All authors contributed to the study conception and design. Material preparation, data collection, and analysis were performed by Autumn Beavers, Elizabeth F. Msoka, and Lily Gutnik. The first draft of the manuscript was written by Autumn Beavers, and all authors commented on previous versions of the manuscript. All authors read and approved the final manuscript.

## Ethics Statement

We obtained ethical approval from the University of Alabama at Birmingham, Kilimanjaro Christian Medical Centre, and the National Institute for Medical Research of Tanzania.

## Consent

Written informed consent was obtained from all individual participants included in the study.

## Conflicts of Interest

The authors declare no conflicts of interest.

## Data Availability

The datasets generated or analyzed during the current study are not publicly available to protect individual privacy but can be obtained from the corresponding author upon reasonable request.
